# Karyotype Evolution and Genomic Organization of Repetitive DNAs in the Saffron Finch, *Sicalis flaveola* (Passeriformes, Aves)

**DOI:** 10.3390/ani11051456

**Published:** 2021-05-19

**Authors:** Rafael Kretschmer, Benilson Silva Rodrigues, Suziane Alves Barcellos, Alice Lemos Costa, Marcelo de Bello Cioffi, Analía del Valle Garnero, Ricardo José Gunski, Edivaldo Herculano Corrêa de Oliveira, Darren K. Griffin

**Affiliations:** 1 School of Biosciences, University of Kent, Canterbury CT2 7NJ, UK; rafa.kretschmer@hotmail.com; 2 Instituto Federal do Pará, Abaetetuba 8440-000, Brazil; benilson.rodrigues@gmail.com; 3 Laboratório de Diversidade Genética Animal, Universidade Federal do Pampa, São Gabriel 97300-162, Brazil; suzianebarcellos@gmail.com (S.A.B.); alicelemoscosta14bio@gmail.com (A.L.C.); analiagarnero@unipampa.edu.br (A.d.V.G.); ricardogunski@unipampa.edu.br (R.J.G.); 4 Centro de Ciências Biológicas e da Saúde, Laboratório de Citogenética de Peixes, Departamento de Genética e Evolução, Universidade Federal de São Carlos, São Carlos 13565-905, Brazil; mbcioffi@ufscar.br; 5 Instituto de Ciências Exatas e Naturais, Universidade Federal do Pará, Belém 66075-110, Brazil; ehco@ufpa.br; 6 Laboratório de Cultura de Tecidos e Citogenética, SAMAM, Instituto Evandro Chagas, Ananindeua 67030-000, Brazil

**Keywords:** Thraupidae, micro and macrochromosomes, inter and intrachromosomal rearrangements, genetic organization, SSRs

## Abstract

**Simple Summary:**

Detailed chromosome studies of birds, addressing both macrochromosomes and microchromosomes, have been reported only for few species. Hence, in this study, we performed investigations of chromosome evolution in the Saffron finch (*Sicalis flaveola*), a semi-domestic species, tolerant of human proximity and nesting in roof spaces. We also explored the organization of simple short repeats (SSR) in the genome of this species. Our results revealed that most of the Saffron finch chromosomes remained highly conserved when compared to the avian ancestral karyotype and that the SSR accumulated mainly in the microchromosomes and the short arms of Z (sex) chromosome. Finally, we compared our results with other avian species, contributing to a better understanding of the chromosome organization and evolution of the Saffron finch genome.

**Abstract:**

The Saffron finch (*Sicalis flaveola*), a semi-domestic species, is tolerant of human proximity and nesting in roof spaces. Considering the importance of cytogenomic approaches in revealing different aspects of genomic organization and evolution, we provide detailed cytogenetic data for *S*. *flaveola*, including the standard Giemsa karyotype, C- and G-banding, repetitive DNA mapping, and bacterial artificial chromosome (BAC) FISH. We also compared our results with the sister groups, Passeriformes and Psittaciformes, bringing new insights into the chromosome and genome evolution of birds. The results revealed contrasting rates of intrachromosomal changes, highlighting the role of SSR (simple short repetition probes) accumulation in the karyotype reorganization. The SSRs showed scattered hybridization, but brighter signals were observed in the microchromosomes and the short arms of Z chromosome in *S*. *flaveola*. BACs probes showed conservation of ancestral syntenies of macrochromosomes (except GGA1), as well as the tested microchromosomes. The comparison of our results with previous studies indicates that the great biological diversity observed in Passeriformes was not likely accompanied by interchromosomal changes. In addition, although repetitive sequences often act as hotspots of genome rearrangements, Passeriformes species showed a higher number of signals when compared with the sister group Psittaciformes, indicating that these sequences were not involved in the extensive karyotype reorganization seen in the latter.

## 1. Introduction

The tanagers (Passeriformes: Thraupidae) exhibit a range of plumage colors and patterns, behaviors, morphologies, and habitats [1]. According to Gill et al. [2], the tanagers are composed of approximately 380 species, representing 4% of the members of the order Passeriformes. Given the extensive diversity found among tanagers, their taxonomic classification has been problematic [1,3]. For instance, the genus *Sicalis* has already been the subject of several taxonomic studies due to controversies on its permanence in Emberizidae [4] or Thraupidae [5]. *Sicalis flaveola*, the subject of this study, is popularly known as the Saffron finch and has an extremely large range in South America [6]. It is a semi-domestic species, tolerant of humans, and frequently nesting in the roof eaves of suburban houses in Eastern Ecuador, Western Peru, Eastern and Southern Brazil (where it is commonly referred to as the “canário-da-terra” or “native canary”—despite not, taxonomically, being a canary).

Cytogenetic studies in tanager species are still scarce and based mostly on conventional staining (Giemsa) [7]. Although only 11% of Thraupidae species have been karyotyped, high chromosomal similarities were observed among them, which approximately 63% of karyotyped species showing 2n = 78 chromosomes [7]. However, some deviations have been described, such as 2n = 72 in *Oryzoborus maximiliani* [8], and 2n = 88 in *Saltator coerulescens* [9]. Molecular cytogenetic studies are even more scarce, with only two species—*Saltator aurantiirostris* and *Saltator similis*, both with 2n = 80—analyzed by comparative chromosome painting using *Gallus gallus* (GGA) and *Leucopternis albicollis* (LAL) probes [10]. Both species presented macrochromosome conservation, except for centric fission of chromosome GGA1, which has been found in all passerines thus far analyzed [11]. 

Despite the low rate of interchromosomal rearrangements in Passeriformes species, a high rate of intrachromosomal rearrangements, such as inversions, have been described, both *in silico* [12,13] and following *in situ* experiments [10,14,15,16,17,18]. The most phylogenetically informative finding is a series of intrachromosomal rearrangements involving paracentric and pericentric inversions in the syntenic group corresponding to GGA1q, including oscines and suboscines [10,14,15,16,17,18]. Therefore, these studies suggested that this complex pattern of intrachromosomal rearrangements was already present in the common ancestor of Passeriformes.

Microchromosomes correspond to approximately 25% of the avian genome [19], and around 50% of avian genes are on these chromosomes [20]. Because of technical limitations, however, most of the molecular cytogenetics studies in Passeriformes have focused only on the comparison of homology with chicken macrochromosomes [11]. For instance, up to now, only four Passeriformes species had their karyotype analyzed in detail, i.e., macro and microchromosomes: *Taeniopygia guttata*, *Turdus merula*, and *Serinus canaria* [17,21,22] from oscines suborder, and *Willisornis vidua* from suboscines suborder [23]. The results revealed that the microchromosomes were not involved in interchromosomal events in the oscines species. However, the chicken microchromosome 17 was found fused to a macrochromosome of *W*. *vidua*. Interchromosomal rearrangements involving these small elements are rare in birds but have been found only extensively in Falconiformes and Psittaciformes [22,24,25,26,27]. In addition, microchromosome fusions have been found in Cuculiformes, Suliformes, and Caprimulgiformes species [28,29], and future studies are necessary to investigate if it is a species-specific feature or if it is shared with other members of these orders. Future studies are also necessary for other Passeriformes members, considering the great diversity in the number of species.

Cytogenomic studies using other types of chromosomal markers, such as repetitive sequences, are also scarce in birds. Repetitive DNA plays an important role in the chromosome structure and genome organization [30,31]. Furthermore, they often serve as hotspots of genome rearrangements and evolutionary innovation [32]. These sequences are classified into distinct categories. Among them are the microsatellites, which represent the most variable types of DNA sequences [33]. To this end, it is essential to know how these elements are organized in the genome. Despite the significance of simple short repetition probes (SSR), data concerning the mapping of these sequences by fluorescent *in situ* hybridization (FISH) are available for a few species of birds and results so far have shown the involvement of amplification of these elements in atypical sex chromosomes, in which the repetitive DNA amount was related to the enlargement of these elements in some cases [34,35,36,37,38,39,40].

The karyotype of *S*. *flaveola* has been investigated only by giemsa staining, revealing a diploid number of 80 chromosomes [41,42,43]. In the present study, we provide the detailed cytogenetic data for the Saffron finch, *S*. *flaveola*, including the standard Giemsa karyotype, C- and G-banding, repetitive DNA mapping, and bacterial artificial chromosome (BAC) FISH, bringing new insights into the chromosome and genome evolution of birds, especially tanagers and Passeriformes.

## 2. Materials and Methods

### 2.1. Animals and Chromosome Preparations

Fibroblast cell lines were established from 1 male and 3 female embryos of *S*. *flaveola*, selected after sexing by Giemsa staining, chromosome banding, and FISH results using BAC for chicken Z and W chromosomes. The cells were cultivated in Dulbecco’s Modified Eagle’s Medium (DMEM) supplemented with 15% fetal bovine serum, 2% penicillin–streptomycin, and 1% L-glutamine at 37 °C, according to Sasaki et al. [44]. Metaphase chromosomes were obtained by standard protocols: treatment with colcemid (1 h), hypotonic solution (0.075 M KCl, 15 min), and fixation with 3:1 methanol/acetic acid. The embryos were collected in their natural environment in São Gabriel city, Rio Grande do Sul State, Brazil, following the procedures approved by the “Biodiversity Authorization and Information System”, permission numbers 44173-1 and 33860-4. The experiments using animals were approved by the Ethics Committee on Animal Experimentation (CEUA) of the Universidade Federal do Pampa under no. 026/2012 and 018/2014.

### 2.2. Diploid Number, C and G-Banding

For the karyotype description and diploid number, an average of 30 metaphases in conventional staining (5% Giemsa in 0.07 M phosphate buffer, pH 6.8) were analyzed per specimen. Chromosomes were arranged and classified according to the nomenclature of Guerra [45]. Blocks of constitutive heterochromatic were detected by C-banding [46]. G-banding patterns were performed according to Schnedl [47], with modifications proposed by Costa et al. [48].

### 2.3. Fluorescence In Situ Hybridization (FISH) with Simple Short Repeat Probes (SSR) and Bacterial Artificial Chromosomes (BAC) Probes

Six simple short repeat probes (SSR) were used: (CA)_15_, (CAA)_10_, (CAC)_10_, (CAG)_10_, (GAA)_10_, and (GAG)_10_. Probes were directly labeled with Streptavidin-Cy3 during their synthesis and the hybridization procedures followed Kubat et al. [49].

A total of 64 bacterial artificial chromosomes (BAC) probes from *G*. *gallus* (GGA, CH261) or *Taeniopygia guttata* (TGMCBA), corresponding to GGA1-28 (except GGA16) and Z and W sex chromosomes were selected and applied to the metaphases of *S*. *flaveola*. Two BAC clones corresponded to pairs GGA4-28 were used (Table S1). However, a higher number of BAC clones were used to pairs GGA1, 2, and 3 in order to detect intrachromosomal rearrangements (Table S1). Isolation, amplification, labeling, and hybridization of BAC clones were performed according to O’Connor et al. [22]. Probes were labeled with Texas red (red) or FITC (green).

### 2.4. Microscopic Analysis and Image Capturing

For conventional experiments, the slides were analyzed using an Olympus DP53 optical microscope. Images of repetitive DNAs FISH experiments were analyzed and captured using a Zeiss Imager 2 microscope with Axiovision 4.8 software (Zeiss, Germany). Images of BAC FISH experiments were captured using a CCD camera and SmartCapture (Digital Scientific UK) system coupled on an Olympus BX61 epifluorescence microscope. Final image processing was performed using Adobe Photoshop 7.0. At least 15 metaphase spreads were analyzed to confirm the chromosomal morphologies and FISH results.

## 3. Results

### 3.1. Karyotype Description, C and G-Banding

**Figure 1:**
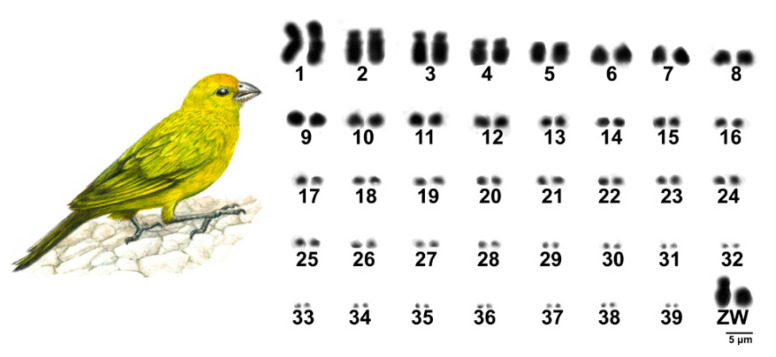
Complete karyotype of a female specimen of *Sicalis flaveola* 2n = 80.

**Figure 2:**
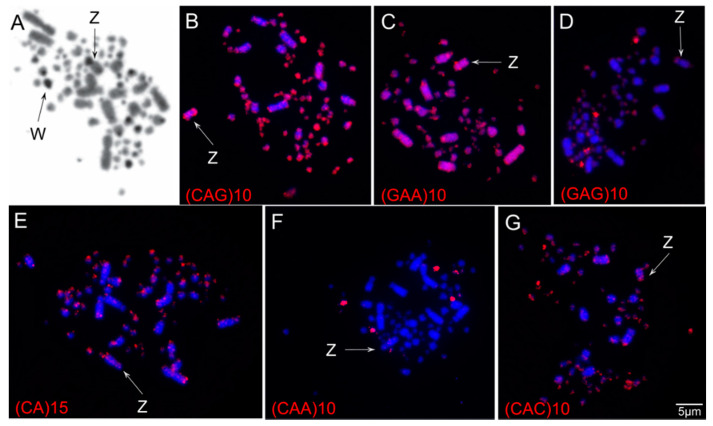
C-banding patterns (**A**) and hybridization of simple short repeats (**B**–**G**) onto metaphases of a female individual of *Sicalis flaveola*. The chromosome probes used are indicated on the left bottom, and the sex chromosomes (Z and W) are indicated by arrows.

The results showed a diploid number of 2n = 80 in *S*. *flaveola*, with 11 pairs of macrochromosomes, including the sex chromosomes, and 28 pairs of microchromosomes, as previously proposed [41,42,43]. Pairs 1, 4, and Z were submetacentric, while the remaining ones were acrocentric (Figure 1). C-banding revealed huge blocks of constitutive heterochromatin in three pairs of microchromosomes, in the short arms of chromosome Z, in the centromere of most macro and microchromosomes, and in the W chromosome, which is heterochromatic in most of its length (Figure 2A).

### 3.2. Chromosomal Distribution of Simple Short Repeats (SSRs)

In general, the SSRs tested here showed scattered hybridization, but a general higher accumulation was observed in the microchromosomes and the short arm of the Z chromosome (Figure 2B–G). The W chromosome, on the other hand, showed dispersed signals, like the autosomes. Specifically, sequences (CA)_15_, (GAA)_10_, (CAG)_10_, and (CAC)_10_ showed scattered signals in all chromosomes but with strong signals on the telomere regions of macrochromosomes and in the microchromosomes (Figure 2B,C,E,G). (GAA)_10_, (CAC)_10_, and (CAG)_10_ also produced signals in the short arms of chromosome Z (Figure 2B,C,G). (GAG)_10_ and (CAA)_10_ produced bright signals in two microchromosome pairs and slight signals in an additional pair of microchromosomes (Figure 2B,F). (GAG)_10_ also showed signals on the Z chromosome (Figure 2B).

### 3.3. Chromosomal Homology Between Chicken and Sicalis flaveola

**Figure 3:**
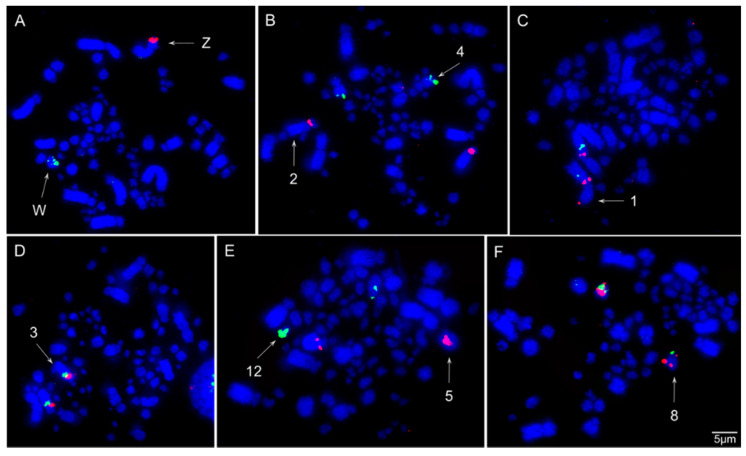
Representative FISH experiments using chicken (CH261) and zebra finch (TGMCBA) macrochromosomes BAC probes in *Sicalis flaveola*: (**A**) chicken macrochromosome Z TGMCBA-270I9 (red) and CH261-94E12 (green); (**B**) chicken macrochromosome 1 TGMCBA-146O14 (red) and TGMCBA-206D5 (green); (**C**) chicken macrochromosome 2 TGMCBA-340P4 (red) and TGMCBA-78C11 (green); (**D**) chicken macrochromosome 3 CH261-130M12 (red) and CH261-97P20 (green); (**E**) chicken macrochromosome 4 CH261-89P6 (red) and CH261-71L6 (green); (**F**) chicken macrochromosome 7 CH261-180H18 (red) and CH261-56K7 (green).

**Figure 4:**
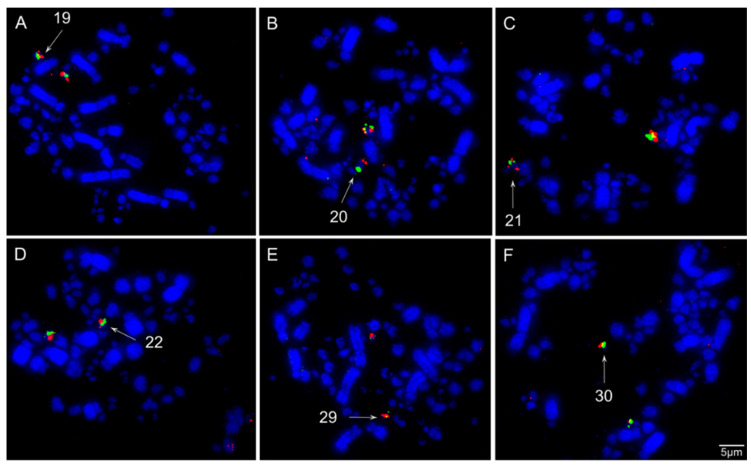
Representative FISH experiments using chicken (CH261) and zebra finch (TGMCBA) microchromosomes BAC probes in *Sicalis flaveola*: (**A**) chicken microchromosome 17 CH261-42P16 (red) and TGMCBA-375I5 (green); (**B**) chicken microchromosome 18 CH261-72B18 (red) and CH26-60N6 (green); (**C**) chicken microchromosome 19 CH261-10F1 (red) and CH261-50H12 (green); (**D**) chicken microchromosome 20 TGMCBA-250E3 (red) and TGMCBA-341F20 (green); (**E**) chicken microchromosome 27 CH261-28L10 (red) and CH261-66M16 (green); (**F**) chicken microchromosome 28 CH261-72A10 (red) and CH261-64A15 (green).

**Figure 5:**
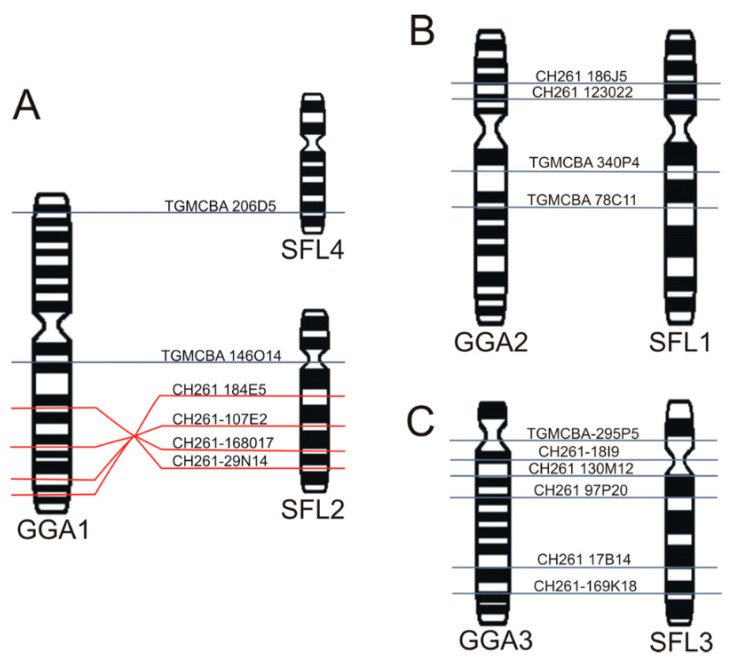
Schematic representation of BAC clones from *Gallus* (CH261) or *Taeniopygia guttata* (TGMCBA) homologous to *G*. *gallus* chromosome 1 (GGA 1) (**A**), chromosome 2 (GGA 2) (**B**), and chromosome 3 (GGA 3) (**C**) in *Sicalis flaveola* (SFL). Ideograms are represented with G-banding patterns. G-banding data from *G*. *gallus* followed Ladjali-Mohammedi et al. [50].

**Figure 6:**
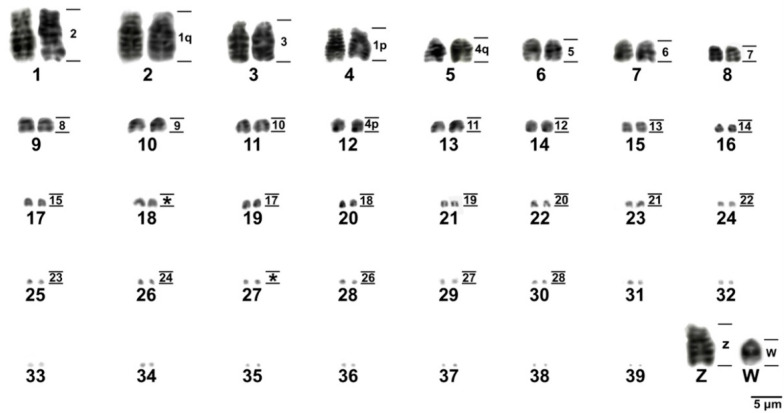
G-banded karyotype of *Sicalis flaveola* and homologous chromosomal segments with *Gallus* chromosomes (right). * Asterisks indicate the probable chromosomes corresponding to GGA16 and GGA25.

The chromosomal mapping of BAC clones corresponding to chicken chromosomes GGA1-28, except 16 and 25, and sex chromosomes Z and W evidenced the syntenic conservation of these chromosomes in *S*. *flaveola* (SFL), with exception of GGA1, which was split into two pairs (SFL 2 and 4) due to centric fission (Figure 3, Figure 4, Figure 5 and Figure 6). The *S*. *flaveola* homologous chromosomes to GGA16 and 25 could not be identified because there were no BAC probes to GGA16, and the probes from GGA25 did not produce signals. Chicken chromosome 4 revealed the GGA4q and 4p as separated chromosomes in *S*. *flaveola* (SFL5 and 12), as in the putative Neognathae karyotype [29]. The analysis of different BAC clones corresponding to GGA1 revealed that intrachromosomal rearrangements occurred in SFL2, homologous to GGA1q. On the other hand, no evidence of this type of rearrangement was observed in the pairs homologous to GGA1p, 2, and 3 (Figure 5). The homology map between *G*. *gallus* and *S*. *flaveola* is shown in Figure 6.

## 4. Discussion

We described here a detailed karyotype description for *S*. *flaveola*, a representative member of the Thraupidae family, and compared our results with previous studies in Passeriformes, especially Thraupidae. Our results confirmed a typical avian karyotype, with 80 chromosomes, divided into 11 pairs of macrochromosomes, including the Z and W sex chromosomes, and 28 pairs of microchromosomes, corroborating the previous karyotype description [41,42,43]. This pattern of karyotype is also typical for Passeriformes and Thraupidae species [7]. 

Despite the constancy of the 2n among Thraupidae species, their C-positive heterochromatin distribution shows distinct patterns among them. In *S*. *flaveola,* we found C-banding positive in three pairs of microchromosomes, in the centromere of the seventh pair, in the W, and in the entire short arms of the Z. Interestingly, in four other species of Thraupidae, *S*. *similis*, *S*. *aurantiirostris*, *Ramphocelus carbo*, and *Tangara cayana*, a block of constitutive heterochromatin was also found in the short arms of the Z chromosome [10,51]. The only exception so far is *Tachyphonus rufus*, in which this block was not found [51]. *R*. *carbo* and *T*. *rufus* are a member from the same subfamily (Tachyphoninae). Hence, it is likely that the block of constitutive heterochromatin is a common trait of Thraupidae family, and it was eliminated in *T*. *rufus*. However, the block of constitutive heterochromatin on the short arms of Z chromosome is not restricted to Thraupidae since a similar pattern has been observed in Passeridae [52,53] and Estrildidae [54] species. Future studies are necessary to investigate if this block of constitutive heterochromatin has a common or independent origin in Thraupidae, Passeridae, and Estrildidae. Nevertheless, these findings highlight that the accumulation/elimination of constitutive heterochromatin in the sex chromosomes is an active process during the chromosomal evolution of Passeriformes. Such role of heterochromatin in the differentiation of sex chromosomes is widely reported in many other groups, including mammals [55], fishes [56,57], plants [58], reptiles [59], among others.

Overall, the SSR probes tested showed scattered hybridization, but brighter signals were observed in the microchromosomes and the short arms of the Z chromosome. They were preferentially associated with heterochromatic regions, corroborating the hypothesis that repetitive DNAs are found in condensed and inactive regions of the genome [11,33]. In addition, previous studies also mentioned that most SSRs are incorporated into non-coding DNA, although they can be found in coding regions, suggesting that these sequences may affect the structure and function of proteins [60].

A distinct pattern of SSRs hybridization has been described in the sex chromosomes among birds. In general, SSRs accumulate in the W chromosomes [28,36,37,38,39], except for Piciformes, in which no specific hybridization signal was observed in this chromosome [35,61]. The Piciformes, in contrast, showed extensive SSRs hybridization signals in the Z chromosome of all species tested so far, which were proposed as the main cause of its enlargement [35,61]. SSRs hybridization signals have also been found in the Z chromosome of Passeriformes and Psittaciformes, but it is not a general rule in species from these orders [36,38]. For instance, in Psittaciformes, it has been found in *Myiopsitta monachus*, but no evidence of SSRs accumulation in *Amazona aestiva* has been observed [36]. Similarly, in Passeriformes, it has been described in *Progne tapera*, but not in *Progne chalybea* and *Pygochelidon cyanoleuca* [38]. Therefore, these studies highlight the role of species-specific repetitive DNAs accumulation in the avian sex chromosomes.

The chromosomal mapping of BAC clones indicated a high degree of inter-chromosomal karyotype conservation between *G*. *gallus* and *S*. *flaveola*, due to the unique interchromosomal rearrangement that was detected, involving centric fission of the ancestral chromosome 1 (GGA1). This fission is widely reported in Passeriformes species and is therefore considered a synapomorphy for the group [11]. Chicken chromosome 4 hybridized two chromosome pairs in *S*. *flaveola* (SFL5 and 12). However, this is the ancestral state, as proposed to the putative Neognathae karyotype [29]. Furthermore, intrachromosomal rearrangements already detected in previous studies in other species of Passeriformes were also observed in the chromosome homologous to GGA1q (SFL2). These paracentric and pericentric inversions occurred in the GGA1q chromosome in different Passeriformes species, both oscines and suboscines [10,15,16,17,18]. Hence, our study reinforces the hypothesis that these intrachromosomal rearrangements were already present in the common ancestral of Passeriformes [15,16].

Most of the cytogenetic studies on birds address only the macrochromosomes, limiting our understanding of the GGA1-9 [11]. Here we provided, for the first time, a detailed analysis of the microchromosomes in Thraupidae species. Our results revealed that the microchromosomes GGA10-28 (except GGA16 that does not have BAC clones, and GGA25, which in turn do not hybridize in Passeriformes species), are conserved as individual chromosomes in *S*. *flaveola*. Similar results were found recently in other oscines species, *Taeniopygia guttata*, *Turdus merula*, and *Serinus canaria* [22]. These data indicate that not only the macrochromosomes but also the microchromosomes are highly conserved among Passeriformes. Thus, we suggest that the ancestral pattern of microchromosome organization was already present in the last common ancestral to Passeriformes. 

**Figure 7:**
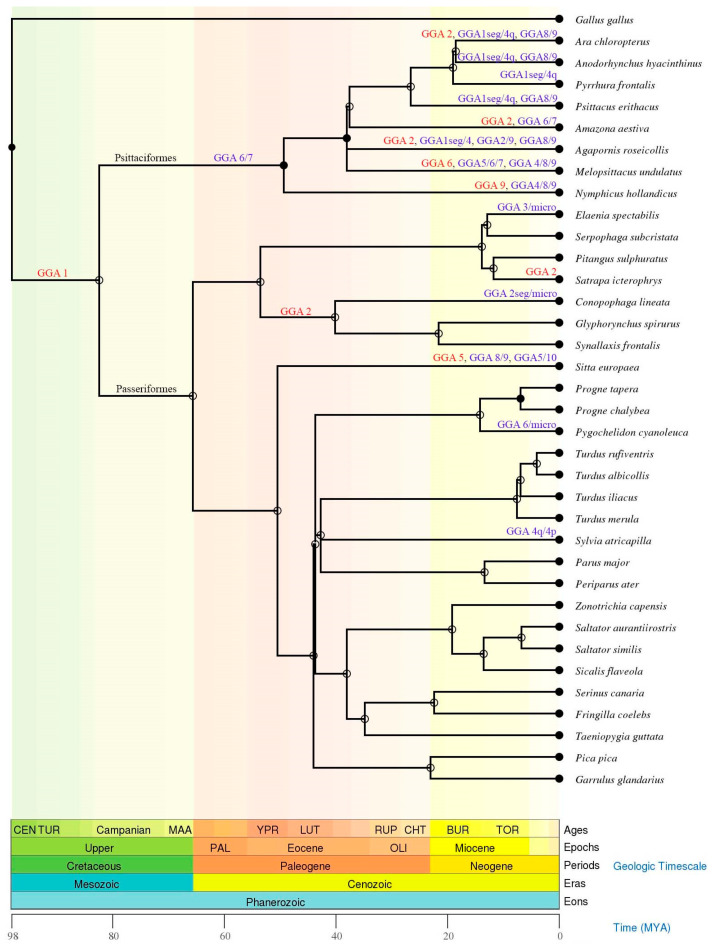
Chromosomal rearrangements in Passeriformes and Psittaciformes species analyzed with chromosome painting with *Gallus gallus* (GGA) probes (GGA1-10) or BACs clones corresponding to these GGA chromosomes. The phylogenetic tree was sourced from TimeTree databases (http://www.timetree.org, accessed on 12 May 2021) [70]. Rearrangements are represented by fissions (red) and fusions (blue). Seg = segment, q = long arm, micro = microchromosome.

The order Passeriformes represents approximately 60% of the avian species [2], and no other avian clade has evolved such great diversity in terms of number of species, morphological and ecological diversification [62]. Interestingly, this diversity was not accompanied by interchromosomal reorganization (Figure 7 and Table S2). On the other hand, parrots (Psittaciformes), the sister group of the Passeriformes [63,64,65], which represent approximately 3.6% of the avian species [2], underwent a high rate of interchromosomal rearrangements, involving fusions of macrochromosomes (Figure 7 and Table S2) and microchromosomes [22,25,27]. This may indicate that the maintenance of the ancestral pattern of karyotype in Passeriformes was crucial to the successful diversification seen in this clade. Intrachromosomal rearrangements, such as inversions, have been extensively described in both Passeriformes and Psittaciformes species. For instance, 125 and 134 intrachromosomal changes have been described in *T*. *guttata* and *Melopsittacus undulatus*, respectively [66]. Although intrachromosomal rearrangements are considered as one of the most prominent adaptation mechanisms [67,68,69], this type of rearrangement does not explain the great difference in terms of the number of species between Passeriformes and Psittaciformes, since both orders underwent a similar amount of intrachromosomal changes.

Repetitive DNA plays an important role in genome organization and function, and they often serve as hotspots of genome rearrangements [31,32]. Hence, by comparing the chromosomal mapping of microsatellite sequences between the sister clades Passeriformes and Psittaciformes, with low and high rates of chromosomal rearrangements, respectively, we can speculate about the importance of these sequence in the karyotype reorganization and the diversification of these clades. Up to now, only four Passeriformes species (*P*. *tapera*, *P*. *chalybea*, *P*. *cyanoleuca*, and *S*. *flaveola*) and two Psittaciformes ones (*Myiopsitta monachus* and *Amazona aestiva*) have been analyzed with chromosomal mapping of SSRs sequences [36,38] (Table 1). Comparing the three SSRs used in common in these species is clear that these Passeriformes have a higher number of signals than the Psittaciformes species. These findings suggest that the SSRs were not involved in the high difference observed between the karyotype organization of Passeriformes and Psittaciformes. However, we cannot discard the involvement of repetitive sequences in the karyotype reorganization of Psittaciformes since other types of these sequences were not explored, such as transposable elements and satellites DNA.

## 5. Conclusions

In the present study, we demonstrated the most complete cytogenetic analysis to date of a Thraupidae family member, contributing to a better understanding of its chromosome organization and evolution. The BAC probes of *G*. *gallus* were applied for the first time in *S*. *flaveola,* showing conservation in the ancestral microchromosomes and most macrochromosomes. Taken together, our findings displayed a typical avian karyotype with a high rate of homology with *G*. *gallus*, some intrachromosomal rearrangements, scattered SSRs distribution, and an uncommon accumulation of these sequences in the Z chromosome. Our comparison of chromosomal mapping of SSRs between the sister clades Passeriformes and Psittaciformes indicated that these sequences were not involved in the karyotype reorganization of Psittaciformes since Passeriformes species showed a higher number of signals.

## Figures and Tables

**Table 1:** Chromosome mapping comparison of microsatellites among Passeriformes and Psittaciformes species.

Species	*SSRs*		
	(CAA)_10_	(CAG)_10_	(CA)_15_
*S*. *flaveola*, 2n = 80 ^1^	Three pairs of micros	Scattered signals in all chromosomes but strong signals on the telomere regions of macros and micros, and in the Zp	Scattered signals in all chromosomes but strong signals on the telomere regions of macros and in the micros
*P*. *cyanoleuca*, 2n = 76 ^2^	Telomere of Wq	Telomere of Wpq	Telomere of Wq
*P*. *tapera*, 2n = 76 ^2^	Telomere of 1q	Wq	Telomere of 1q, 2q, Wq; Pericentromeric region of 6, 7, Wq
*P*. *chalybea*, 2n = 76 ^2^	Telomere of 1pq, 2q, Wq; pericentromeric region of 1pq	-	Telomere of 1pq, 2q, 4q, Wq; pericentromeric region of 1pq, Wpq
*M*. *monachus*, 2n = 48 ^3^	Wq	Telomere region of 1p, 2q, 3q, 4pq, 5q, 6p, Zp; pericentromeric region of 1q, 7q, Wq; centromeric region 1-9; all micros	-
*A*. *aestiva*, 2n = 70 ^3^	-	-	-

^1^ Present study, ^2^ Barcellos et al. [38], ^3^ Furo et al. [36], 2n = diploid number, macro = macrochromosomes, micro = microchromosomes, p = short arms, and q = long arms.

## Data Availability

The data presented in this study are available in the article or supplementary material.

## Supplementary Materials

The following are available online at https://www.mdpi.com/article/10.3390/ani11051456/s1, Table S1: List of BACs applied to *Sicalis flaveola* (SFL); and Table S2: List of Passeriformes and Psittaciformes species analyzed with *Gallus gallus* (GGA) chromosome painting or BACs clones corresponding to GGA1-10.
