# Supplementary material for: Karyotype Evolution and Genomic Organization of Repetitive DNAs in the Saffron Finch, Sicalis flaveola (Passeriformes, Aves)
Source: Animals (Basel). 2021 May 19;11(5):1456. doi: 10.3390/ani11051456 (PMC8160697; doi:10.3390/ani11051456)
Supplement: Supplementary file 1 [file animals-11-01456-s001.zip › Table S2.pdf]

Supplementary Table 2 - List of Passeriformes and Psittaciformes species analyzed with *Gallus gallus* (GGA) chromosome painting or BACs clones corresponding to GGA1-10.

Species	Family	2n[1]	Fusions	Fissions	References
			**Passeriformes**		
Conopophaga lineata	Conopophagidae	78	GGA2seg[2]/micro[3]	GGA1=CLI2 and 7<br>GGA2=CLI1 and 5	[1]
Pica pica	Corvidae	76	-	GGA1=PPI3 and 4	[2]
Garrulus glandarius	Corvidae	78	-	GGA1=GGL2 and 4	[2]
Taeniopygia guttata	Estrildidae	80	-	GGA1=TGU2 and 5	[3]
Serinus canaria	Fringillidae	80	-	GGA1=SCA2 and 5	[3]
Fringilla coelebs	Fringillidae	80	-	GGA1=FCO3 and 4	[4]
Synallaxis frontalis	Furnariidae	82	-	GGA1=SFR1 and 5<br>GGA2=SFR3 and 7	[5]
Glyphorynchus spirurus	Furnariidae	80	-	GGA1=GSP3 and 4<br>GGA2=GSP2 and 5	[6]
Progne chalybea	Hirundinidae	76	-	GGA1=PCH2 and 5	[7]
Progne tapera	Hirundinidae	76	-	GGA1=PTA2 and 5	[7]
Pygochelidon cyanoleuca	Hirundinidae	76	GGA6/micro	GGA1=PCY2 and 5	[7]
Zonotrichia capensis	Passerellidae	80	-	GGA1=ZCA2 and 5	[8]
Parus major	Paridae	80	-	GGA1=PMA2 and 4	[2]
Periparus ater	Paridae	80	-	GGA1=PAT2 and 4	[2]
Sitta europaea	Sittidae	80	GGA8/9=SEU7<br>GGA5/10=SEU6	GGA1= SEU2 and 5<br>GGA5=SEU6q and 10	[2]
Sylvia atricapilla	Sylviidae	74	GGA4q/GGA4p=SAT5	GGA1=SAT2 and 4	[2]
Saltator aurantiirostris	Thraupidae	80	-	GGA1=SAU2 and 5	[9]
Saltator similis	Thraupidae	80	-	GGA1=SSI2 and 5	[9]
Sicalis flaveola	Thraupidae	80	-	GGA1=SFL2 and 4	Present study
Turdus rufiventris	Turdidae	78	-	GGA1=TRU2 and 5	[10]
Turdus albicollis	Turdidae	78	-	GGA1=TAL2 and 5	[10]
Turdus iliacus	Turdidae	80	-	GGA1=TIL2 and 5	[4]

Turdus merula	Turdidae	80	-	GGA1=TME2 and 5	[11]
Elaenia spectabilis	Tyrannidae	80	-	GGA1=ESP2 and 5	[12]
Pitangus sulphuratus	Tyrannidae	80	-	GGA1=PSU3 and 5	[13]
Serpophaga subcristata	Tyrannidae	82	-	GGA1=SSU3 and 5	[13]
Satrapa icterophrys	Tyrannidae	82	-	GGA1=SIC2 and 5	[13]
			**Psittaciformes**		
Nymphicus hollandicus	Cacatuidae	72	GGA6/7=NHO5<br>GGA4/8/9=NHO4	GGA1=NHO3 and 6<br>GGA9=NHO4p and 10	[14]
Ara chloropterus	Psittacidae	70	GGA1seg/4q=ACH1<br>GGA6/7=ACH6<br>GGA8/9=ACH7	GAA1=ACH1q and 4<br>GGA2=ACH2 and 11	[15]
Anodorhynchus hyacinthinus	Psittacidae	70	GGA1seg/4q=AHY1<br>GGA6/7=AHY6<br>GGA8/9=AHY7	GGA1=AHY1q and 4	[15]
Psittacus erithacus	Psittacidae	62-64	GGA1seg/4=PER1<br>GGA6/7=PER6<br>GGA8/9=PER7	GGA1=PER1q and 4	[16]
Ara macaw	Psittacidae	62-64	GGA1seg/4q=AMA1<br>GGA6/7=AMA6<br>GGA8/9=AMA7	GGA1=AMA1, 4, and 9	[17]
Pyrrhura frontalis	Psittacidae	70	GGA1q/4=PFR1<br>GGA6/7=PFR6	GGA1=PFR1q and 4	[18]
Amazona aestiva	Psittacidae	70	GGA6/7=AAE7	GGA1=AAE2 and 5<br>GGA2=AAE1 and 12	[18]
Agapornis roseicollis	Psittaculidae	48	GGA6/7=ARO6<br>GGA1/4=ARO4<br>GGA8/9=ARO5<br>GGA2/9=ARO9	GGA1=ARO3 and 4q<br>GGA2=ARO2 and 9q<br>GGA9=ARO5q and 9q	[14]
Melopsittacus undulatus	Psittaculidae	62	GGA5/6/7=MUN4<br>GGA4/8/9=MUN5	GGA1=MUN3 and 6<br>GGA6=MUN4p and 8p	[14]

[1] Diploid number, [2] segment, and [3] microchromosome. The data of *Myiopsitta monachus* (Psittaciformes) were not compared here because the exact fusions involving chromosomes GGA10, 11, and 12 were not identified, since a flow sorted peak including the chromosomes GGA10, 11, and 12 was used [19]. The data of *Willisornis vidua* (Passeriformes) were not compared here because the authors reported several interchromosomal rearrangements in this species, however, the exact rearrangements were not described [20].

References

1. de Oliveira, T.D.; Kretschmer, R.; Bertocchi, N.A.; O'Brien, P.C.M.; Ferguson-Smith, M.A.; Garnero, A.D.V.; de Oliveira, E.H.C.; Gunski, R.J. The molecular cytogenetic characterization of *Conopophaga lineata* indicates a common chromosome rearrangement in the Parvorder Furnariida (Aves, Passeriformes). *Genet*. *Mol*. *Biol*. **2020**, *43*, e20200018. doi: 10.1590/1678-4685-gmb-2020-0018
2. Nanda, I.; Benisch, P.; Fetting, D.; Haaf, T.; Schmid, M. Synteny conservation of chicken macrochromosomes 1-10 in different avian lineages revealed by cross-species chromosome painting. *Cytogenet*. *Genome Res*. **2011**, *132*, 165–181. doi: 10.1159/000322358
3. dos Santos, M.S.; Kretschmer, R.; Frankl-Vilches, C.; Bakker, A.; Gahr, M.; O'Brien, P.C.M.; Ferguson-Smith, M.A.; de Oliveira, E.H.C. Comparative cytogenetics between two important songbird, models: The zebra finch and the canary. *PLoS One* **2017**, *12*, e0170997. doi: 10.1371/journal.pone.0170997
4. Derjusheva, S.; Kurganova, A.; Habermann, F.; Gaginskaya, E. High chromosome conservation detected by comparative chromosome painting in chicken, pigeon and passerine birds. *Chromosome Res*. **2004**, *12*, 715-723. doi: 10.1023/B:CHRO.0000045779.50641.00
5. Kretschmer, R.; de Lima, V.L.C.; de Souza, M.S.; Costa, A.L.; O'Brien, P.C.M.; Ferguson-Smith, M.A.; de Oliveira, E.H.C.; Gunski, R.J.; Garnero, A.D.V. Multidirectional chromosome painting in *Synallaxis frontalis* (Passeriformes, Furnariidae) reveals high chromosomal reorganization, involving fissions and inversions. *Comp*. *Cytogenet*. **2018**, *12*, 97-110. doi: 10.3897/CompCytogen.v12i1.22344
6. Ribas, T.F.A.; Nagamachi, C.Y.; Aleixo, A.; Pinheiro, M.L.S.; O´Brien, P.C.M.; Ferguson-Smith, M.A.; Yang, F.; Suarez, P.; Pieczarka, J.C. Chromosome painting in *Glyphorynchus spirurus* (Vieillot, 1819) detects a new fission in Passeriformes. *PLoS One* **2018**, *13*, e0202040. doi: 10.1371/journal.pone.0202040
7. Barcellos, S.; Kretschmer, R.; de Souza, M.S.; Costa, A.L.; Degrandi, T.M.; dos Santos, M.S.; de Oliveira, E.H.C.; Cioffi, M.B.; Gunski, R.J.; Garnero, A.V. Karyotype Evolution and Distinct Evolutionary History of the W Chromosomes in Swallows (Aves, Passeriformes). *Cytogenet*. *Genome Res*. **2019**, *158*, 98–105. doi: 10.1159/000500621
8. Bülau, S.E.; Kretschmer, R.; Gunski, R.J.; Garnero, A.D.V.; O'Brien, P.C.M.; Ferguson-Smith, M.A.; de Oliveira, E.H.C.; de Freitas, T.R.O. Chromosomal polymorphism and comparative chromosome painting in the rufous-collared sparrow (*Zonotrichia capensis*). *Genet*. *Mol*. *Biol*. **2018**, *41*, 799–805. doi: 10.1590/1678-4685-gmb-2017-0367
9. dos Santos, M.S.; Kretschmer, R.; Silva, F.A.O.; Ledesma, M.A.; O'Brien, P.C.M.; Ferguson-Smith, M.A.; Garnero, A.D.V.; de Oliveira, E.H.C.; Gunski, R.J. Intrachromosomal rearrangements in two representatives of the genus *Saltator* (Thraupidae, Passeriformes) and the occurrence of heteromorphic Z chromosomes. *Genetica* **2015**, *143*, 535-543. doi: 10.1007/s10709-015-9851-4
10. Kretschmer, R.; Gunski, R.J.; Garnero, A.D.V.; Furo, I.O.; O'Brien, P.C.M.; Ferguson-Smith, M.A.; de Oliveira, E.H.C. Molecular cytogenetic characterization of multiple intrachromosomal rearrangements in two representatives of the genus *Turdus* (Turdidae, Passeriformes). *PLoS One* **2014**, *9*, e103338. doi: 10.1371/journal.pone.0103338
11. Guttenbach, M.; Nanda, I.; Feichtinger, W.; Masabanda, J.S.; Griffin, D.K.; Schmid, M. Comparative chromosome painting of chicken autosomal paints 1–9 in nine different bird species. *Cytogenet*. *Genome Res*. 2003, 103, 173-184. doi: 10.1159/000076309
12. Kretschmer, R.; de Oliveira, E.H.C.; dos Santos, M.S.; Furo, I.O.; O'Brien, P.C.M.; Ferguson-Smith, M.A.; Garnero, A.D.V.; Gunski, R.J. Chromosome mapping of the large elaenia (*Elaenia spectabilis*): Evidence for a cytogenetic signature for passeriform birds? *Biol*. *J*. *Linn*. *Soc*. **2015**, *115*, 391-398. doi: 10.1111/bij.12504
13. Rodrigues, B.S.; Kretschmer, R.; Gunski, R.J.; Garnero, A.D.V.; O'Brien, P.C.M.; Ferguson-Smith, M.A.; de Oliveira, E.H.C. Chromosome Painting in Tyrant Flycatchers Confirms a Set of Inversions Shared by Oscines and Suboscines (Aves, Passeriformes). *Cytogenet*. *Genome Res*. **2018**, *153*, 205-212. doi: 10.1159/000486975

14. Nanda, I.; Karl, E.; Griffin, D.K.; Schartl, M.; Schmid, M. Chromosome repatterning in three representative parrots (Psittaciformes) inferred from comparative chromosome painting. *Cytogenet. Genome Res.* **2007**, *117*, 43-53. doi: 10.1159/000103164
15. Furo, I.O.; Kretschmer, R.; O'Brien, P.C.M.; Ferguson-Smith, M.A.; de Oliveira, E.H.C. Chromosomal Diversity and Karyotype Evolution in South American Macaws (Psittaciformes, Psittacidae). *PLoS One* **2015**, *10*, e0130157. doi: 10.1371/journal.pone.0130157
16. Seibold-Torres, C.; Owens, E.; Chowdhary, R.; Ferguson-Smith, M.A.; Tizard, I.; Raudsepp, T. Comparative Cytogenetics of the Congo African Grey Parrot (*Psittacus erithacus*). *Cytogenet. Genome Res.* 2015, 147, 144–153. doi: 10.1159/000444136
17. Seabury, C.M.; Dowd, S.E.; Seabury, P.M.; Raudsepp, T.; Brightsmith, D.J.; Liboriussen, P.; Halley, Y.; Fisher, C.A.; Owens, E.; Viswanathan, G.; Tizard, I.R. A Multi-Platform Draft de novo Genome Assembly and Comparative Analysis for the Scarlet Macaw (*Ara macao*). *PLoS One* **2013**, *8*, e62415. doi: 10.1371/journal.pone.0062415
18. Furo, I.O.; Kretschmer, R.; O'Brien, P.C.M.; Pereira, J.C.; Garnero, A.D.V.; Gunski, R.J.; Ferguson-Smith, M.A.; de Oliveira, E.H.C. Chromosome painting in neotropical long and short-tailed parrots (Aves, Psittaciformes): phylogeny and proposal for a putative ancestral karyotype for tribe Arini. *Genes* **2018**, *9*, 491. doi: 10.3390/genes9100491
19. Furo, I.O.; Kretschmer, R.; O'Brien, P.C.M.; Pereira, J.; Garnero, A.D.V.; Gunski, R.J.; O'Connor, R.E.; Griffin, D.K.; Gomes, A.J.B.; Ferguson-Smith, M.A.; de Oliveira, E.H.C. Chromosomal evolution in the phylogenetic context in Neotropical Psittacidae with emphasis on a species with high karyotypic reorganization (*Myiopsitta monachus*). *Front. Genet.* **2020**, *11*, 721. doi: 10.3389/fgene.2020.00721
20. Ribas, T.F.A.; Pieczarka, J.C.; Griffin, D.K.; Kiazim, L.G.; Nagamachi, C.Y.; O´Brien, P.C.M.; Ferguson-Smith, M.A.; Yang, F.; Aleixo, A.; O'Connor, R.E. Analysis of multiple chromosomal rearrangements in the genome of *Willisornis vidua* using BAC-FISH and chromosome painting on a supposed conserved karyotype. *BMC Ecol. Evol.* **2021**, *21*, 34. doi: 10.1186/s12862-021-01768-y
